# Tactile Biography Questionnaire: A contribution to its validation in an Italian sample

**DOI:** 10.1371/journal.pone.0274477

**Published:** 2022-09-15

**Authors:** Isabella Lucia Chiara Mariani Wigley, Massimiliano Pastore, Eleonora Mascheroni, Marta Tremolada, Sabrina Bonichini, Rosario Montirosso

**Affiliations:** 1 Department of Developmental and Social Psychology, University of Padua, Padua, Italy; 2 0–3 Center for the at-Risk Infant, Scientific Institute IRCCS Eugenio Medea, Bosisio Parini, Lecco, Italy; University of Pavia: Universita degli Studi di Pavia, ITALY

## Abstract

As the interest in the beneficial effects of positive touch experiences is rapidly growing, having reliable and valid tools to its assessment is essential. The Tactile Biography Questionnaire (TBQ) allows to quantify individual differences in affective touch experiences throughout life. The aim of this article is to present a contribution to its validation in the Italian population. Data analysis were run on a sample of 2040 Italian individuals (Females = 1342, 64%) participating in an on-line survey. Confirmatory Factor Analysis and invariance analyses for gender were applied. Concurrent validity was checked using two specific subscales of the Touch Avoidance Questionnaire (TAQ—i.e., *Family* and *Stranger*). The four-dimensional structure of the TBQ was confirmed in the overall sample and by gender. Also, the TBQ showed an excellent internal consistency and a good concurrent validity with TAQ. The present study suggests that the TBQ can be used to support healthcare professionals and researchers to assess experiences of affective touch in different settings.

## Introduction

Interpersonal affective touch is a crucial factor in mammals development [1]. Studies conducted on non-human animals highlighted the importance of early caring maternal behaviors such as licking and grooming, which affect later-life outcomes on brain maturation through several processes including epigenetic mechanisms [2]. Similarly, the physical expression of affection in humans plays a key role from the first moments of life and throughout development, featuring a preferential neurophysiological pathway to be processed [3–8].

Beneficial effects of affective touch on neurophysiological regulation and behavioral outcomes have been identified throughout the lifespan. At birth, affective touch stimulation reduces physical arousal and heart rate in healthy infants [9, 10]. Even in an at-risk condition such as prematurity, gentle-touch significantly improves physiological regulation [11], and promotes early affiliative behaviors and parent-infant bonding [12]. During childhood, the frequency of affective touch enacted with parents and siblings was associated with the strength of connectivity of the posterior superior temporal sulcus and other nodes of the social brain [13] and was predictive of children’s expression of positive emotions [14]. In adolescence and adulthood, affective touch appears in new facets such as those related to romantic and sexual attraction. However, the effects of touch in this phase of life are not limited to these aspects. Non-romantic affective touch provided by a stranger experimenter activates brain circuits involved in social cognition and reward, which is not the case with non-affective touch stimulation provided by the same experimenter [15]. In the light of this evidence, individual exposure to affective touch is associated with different aspects of well-being throughout the life span.

In the last decades, studies regarding touch have increased dramatically and include different methodologies of investigation such as the assessment of physiological responses to tactile stimulation directly [16], psychophysical protocols for controlled stimulus delivery (e.g., robotic tactile stimulation technique) [17–20] and observational scales which allow to code presence/absence as well as type of touch-related behaviors observed [21–25].

Although studies investigating touch through psychometric tools such as questionnaires and self-reports are growing [26, 27], there is a scarcity of validated and reliable psychometric tools to assess behavior, attitudes, and experiences related to touch. Well-constructed self-report scales can predict a wide range of important outcomes with only relative efficiency and ease [28]. For this reason, establishing a psychometric tools that assess individual differences in the domain of affective touch is needed. Recently, for example, to overcome this methodological gap, a self-rating instrument for quantifying individual exposure of affective tactile experiences was developed by Beltran and colleagues [29]. The Tactile Biography Questionnaire (TBQ) consists of 28 items grouped in 4 different components: *Childhood/Adolescent Touch Experience*, *Comfort with Interpersonal Touch*, *Fondness for Interpersonal Touch*, *Adult Touch Experience*. The TBQ has shown to have excellent internal consistency and good construct validity [29]. Its good psychometric properties and the limited number of items make the TBQ a reliable and useful tool to use when assessing individual differences in tactile biography.

In the Italian landscape, there are no validated questionnaires designed at exploring individual differences in affective touch experiences. Therefore, the Italian validation of TBQ would be a useful contribution for national studies and could open new perspectives for multidisciplinary research on the role of affective touch in different settings.

The main objectives of the present study were to test the factorial structure of the TBQ [29] and to provide new insights about the psychometric properties of this questionnaire in an Italian sample. First, the study aimed to test the factorial structure of the TBQ originally proposed by the Authors (S1 Fig) using a confirmatory analysis with a two-steps analytic approach. Second, as several studies have highlighted differences between males and females in the perception of affective touch (e.g., females seem to be more sensitive to affective touch as well as to discriminative aspects of touch) [30], in the present study we tested the measurement invariance of the TBQ according to gender. Third, the concurrent validity of the TBQ was tested. Finally, as the data collection was performed during the coronavirus pandemic, information about the impact of *COVID-19* on participants and relatives was collected in order to control for possible effects on touch-related factors.

## Materials and methods

### Participants and procedure

Data were collected between May and August 2021, using an on-line anonymous survey setup with *Qualtrics*. Participants’ inclusion criteria were a) be older than 18 years old and b) be an Italian native speaker. All participants were recruited through snowball sampling and an informed written consent was obtained. The survey was filled by 2612 individuals (Females = 1478; 70%). Participants with data missing from more than 25% of items were eliminated from subsequent analyses. Participants who agreed to participate and completed the 85% of items but did not fulfill the eligibility criteria were excluded from the final sample. The final sample included 2040 participants (Females = 1342; 64%) and their descriptive statistics are resumed in Table 1. The present sample size resulted more than acceptable ensuring that the N/p ratio (where N is the number of subjects and p is the number of indicator variables) is above 10 [31].

**Table 1:** Descriptive statistics and *COVID-19* related variables.

Participants’ characteristics (N = 2040)		
Age (range 18–65 yrs)	M = 30.96 yrs SD = 13.86	
	N	
Gender (N = 1976)		
Females	1342	
Males	608	
Not declared	26	
Marital status (N = 1896)		
*Married/ Cohabitant*	549	
*Divorced/Separated*	79	
*Single*	1264	
Not declared	94	
Education (N = 2010)		
*Elementary school*	5	
*Middle school*	140	
*High school*	1358	
*Bachelor degree*	429	
*PhD/Master*	78	
*COVID-19* related variables		
Fear of *COVID-19* (range 0–10)	M = 5.73 SD = 2.27	
	N	
	Yes	No
Tested positive for *COVID-19* (self)	231	1805
Tested positive for *COVID-19* (relatives)	1200	836
Grief for *COVID-19* (someone close)	203	1831

Descriptive statistics of included and excluded participants were compared for gender, age and *COVID-19* related variables and no differences were found (S2 Fig). The entire procedure was reviewed and approved by the University of Padua ethics panel (number of protocol: 6726ADCF521BE52EDBF4DC6C4A0485B9). Recruiting and testing conformed with the local Ethics Committee requirements and the Declaration of Helsinki.

### Translation of the original version of the Tactile Biography Questionnaire

The original version of the TBQ was translated from English to Italian by two of the authors of the present work [ILCMW, EM]. The Italian translation was then back-translated by a professional translator practiced in both languages and revised to solve possible conceptual differences in items contents. To ensure a good level of item equivalence, original and back-translated versions were compared by an author of the original version of the TBQ [MB] [29]. The Italian version of the questionnaire is included in S1 Table.

### Measures

#### Sample demographics and COVID-19 related variables

The socio-demographic section included questions asking general information (i.e., age, gender, nationality, civil status, educational level). As the data collection was performed during *COVID-19* pandemic, a specific section of the survey was devoted to collect information about the impact of *COVID-19* situation on participants and relatives using the following yes/no questions: 1. “*have you ever tested positive for COVID-19*?”, 2. “*have some of your relatives ever tested positive for COVID-19*?”, 3. “*have you lost someone close to you because of COVID-19*?”. In addition, the last question 4. “*how scared are you of COVID-19*?” could be answered on a rating-scale ranging from 0 (at all) to 10 (a lot), where higher score reflects higher level of fear of *COVID-19*.

#### Touch Avoidance Questionnaire

In order to test TBQ’s concurrent validity, the Touch Avoidance Questionnaire (TAQ) was administered. The psychometrics properties of its Italian version have been previously reported [32, 33]. The TAQ was developed in order to assess the level of touch avoidance in different contexts, such as situations involving romantic partner, parents and strangers [33]. The TAQ consists of 31 Likert-type items to which participants are asked to respond on a five-point scale (1 “fully disagree”; 5 “fully agree”) where higher scores reflect higher level of touch avoidance. Items are divided in 5 subscales (*Partner*, *Family*, *Same sex*, *Opposite-sex* and *Stanger*) obtained by averaging relevant items. For the purposes of the present study, only *Family* (six items) and *Stranger* (three items) subscales were selected. *Family* subscale includes items such as “*I grew up in a cuddly family*” while *Stranger* subscale comprises statements such as “*I find it very unpleasant to be in contact with unknown people*”. Here, McDonald’s Omegas were .80 and .51 for *Family* and *Stranger* subscales, respectively. McDonald’s Omega of the *Stranger* subscale was relatively low, but it was in line with internal consistency values reported in the Italian validation study [32].

#### Tactile Biography Questionnaire

Participants’ touch experience was assessed using the TBQ [29]. The questionnaire is composed of 29 items rated on a 5-point Likert scale (ranging from 1 “Never*”* to 5 “Very frequently*”*). The respondent is asked to indicate how frequent the stated event occurred or the degree to which each statement applied to them. Items are grouped in 4 different domains: 1. *Childhood/Adolescent Touch Experience*, 10 items (e.g., “*As a child I received affective touch from family members (parents/caregivers”)*; 2. *Comfort with Interpersonal Touch*, 6 items (e.g., “*How comfortable do you feel with caressing/stroking touch in close (romantic and non-romantic) relationships*”); 3. *Fondness for Interpersonal Touch*, 5 items (e.g., “*I have always liked to receive caresses from someone that I am close to*”); 4. *Adult Touch Experience*, 7 items (e.g., “*In my adult life I have given affective touch to close friends or family members*”). Additionally, three multiple-answer items were also included in the questionnaire asking: 1. “*Which emotion is generated by affective touch in close relationships*”, 2. “*Personal preference to give or receive affective touch in close relationships*” and, 3. “*The presence of negative or unpleasant experiences involving interpersonal touch*”. With the first multiple-answer item, participants could identify which emotion is generated by affective touch in close interpersonal relationships from a range of emotions (5 with a positive value, 5 with a negative value). The second multiple-answer item requires the participant to indicate an historic preference to give or receive affective touch in close relationships. Finally, the last multiple-answer item consists of a yes/no question in which the participants could indicate whether they recognized in their personal history the presence of negative or unpleasant experiences involving interpersonal touch. Participants can indicate the type of experience motivating the answer. The original study reported good psychometric properties of the TBQ with Cronbach’s α values ranging from 0.73 to 0.94 [29]. Here, McDonald’s Omegas were from 0.77 to 0.88.

### Plan of analysis

A cross validation with a two-step analytic approach was carried out. The original sample was split into two independent randomly chosen sub-samples, the calibration sample, which included *N*_*c*_ = 1246 subjects, and the validation sample, which included *N*_*v*_ = 749 subjects. The two samples did not differ as far as age, gender and *COVID-19* related variables (S3 Fig). The observed-variables model was developed using the calibration data sample (first step) and then confirmed using the independent validation sample (second step).

In the first step (calibration sample), we explored items’ distribution using a visual representation and then evaluated the factorial properties of the TBQ. A Confirmatory Factor Analysis (CFA) using robust diagonally weighted least squares (DWLS) for ordinal items (e.g., Likert-type scales) was used to test the structure of the scale originally proposed by the Authors [29] (S1 Fig). In accordance with the analysis plan proposed by the authors of the original version of the TBQ, items number 17, 30, 31 and 32 were excluded from CFA [29]. To evaluate the overall model fit, the following indices were used: comparative fit index (CFI), non-normed fit index (NNFI; also known as the Tucker-Lewis index-TLI), root mean square error of approximation (RMSEA) [90% confidence interval (CI)], the SRMR (Standardized Root Mean Square Residual), TCD (Total Coefficient of Determination) and McDonald’s Omega. The CFI and NNFI indicate an optimal fit when their values are greater than 0.95 while the cut-off suggested for RMSEA and SRMR are respectively 0.08 and 0.06 [34]. TCD estimates the amount of explained model variance and ranges from 0 (i.e., 0% of variance explained) to 1 (i.e., 100%) such that the closer it is to 1, the better the fit [35]. In addition, sensitivity analysis for Structural Equation Modeling was carried out in order to evaluate the presence of influential cases [36].

In the second step (validation sample), we re-tested the structural model, and we compared the fit indices and parameter estimates derived from the validation and calibration samples.

In order to examine measurement invariance of the TBQ according to gender (males and females), a multi-group CFA was performed. A hierarchical approach was considered by successively constraining model parameters (i.e., factor loadings, thresholds and residual variances) and comparing changes in model fit. Four models were estimated (i.e., configural, factor loadings, thresholds and residual) and represented prerequisites for meaningful across-group comparisons based on factor scales, following a scheme suggested by Bowen and Masa [37]. As the use of *Δχ*2 values has been criticized because of their sensitivity to sample size [38], testing for invariance was examined through the practical perspective [39], which recommends that invariance can be based on two criteria: (a) the multigroup factor model exhibits an adequate fit to the data and (b) the change in values for fit indices (e.g., ΔCFI, ΔRMSEA) is negligible. A ΔCFI larger than .01 and a change larger than .015 in ΔRMSEA is indicative of non-invariance [40–42]. Moreover, in order to evaluate invariance between males and females, a bootstrap approach was also used. This approach allows us to estimate the sampling distribution of a statistic of interest (e.g., factor loadings) by resampling with replacement from the original sample without normality assumption [43]. In the current study, we graphically represented the distributions of factor loadings and computed the proportion of overlapping across paired items (males and females). We created 5000 bootstrapped replicates sampling from the original data in order to ensure that the actual number of acceptable solutions (*N* = 3279) was sufficiently large, extracted the empirical distribution of each factor loading, and evaluated the overlapping area for each corresponding pair of items (e.g., item 1 for males and item 1 for females).

In order to examine the concurrent validity of the TBQ, bivariate correlations were performed between *Family* and *Strangers* TAQ subscales and TBQ factor scores (i.e., estimated values for the latent variables).

Finally, possible associations between TBQ factor scores (i.e., estimated values for the latent variables) and *COVID-19* related variables were tested in order to control for possible effects of the pandemic situation on the individual tactile experiences reports.

All data analyses were performed with the R statistical software (R Core Team, 2015) and using lavaan [44], influence.SEM [45] and Overlapping [46] packages for CFAs, measurement invariance and overlapping analysis respectively.

## Results

### First step: Confirmatory factor analysis in the calibration sample

A graphical representation of the percentages of TBQ items scores in the calibration sample (*N*_*c*_ = 1246) is displayed in S4 and S5 Figs.

The results of the CFA revealed high and homogenous items’ loadings that were between 0.40 and 0.91 (S2 Table). All the fit indices (CFI, NNFI, RMSEA, SRMR, TCD) obtained in the calibration sample are reported in Table 2.

**Table 2:** Fit indices in the calibration and validation sample.

	CFI	NNFI	RMSEA	SRMR	TCD
Calibration Sample	0.970	0.967	0.102	0.077	0.999
Validation Sample	0.974	0.971	0.095	0.073	0.999

CFI = comparative fit index; NNFI = Tucker–Lewis index; RMSEA = root mean square error of approximation; SRMR = Standardized root mean square residual. TCD = total coefficient of determination.

Given the low percentage of missing data (S6 Fig), a listwise deletion strategy was performed, excluding subjects with missing values (N = 78) from the analyses. In S3a and S3b Table, results obtained by using a full information maximum likelihood approach for imputing missing data are reported.

**Fig 1:**
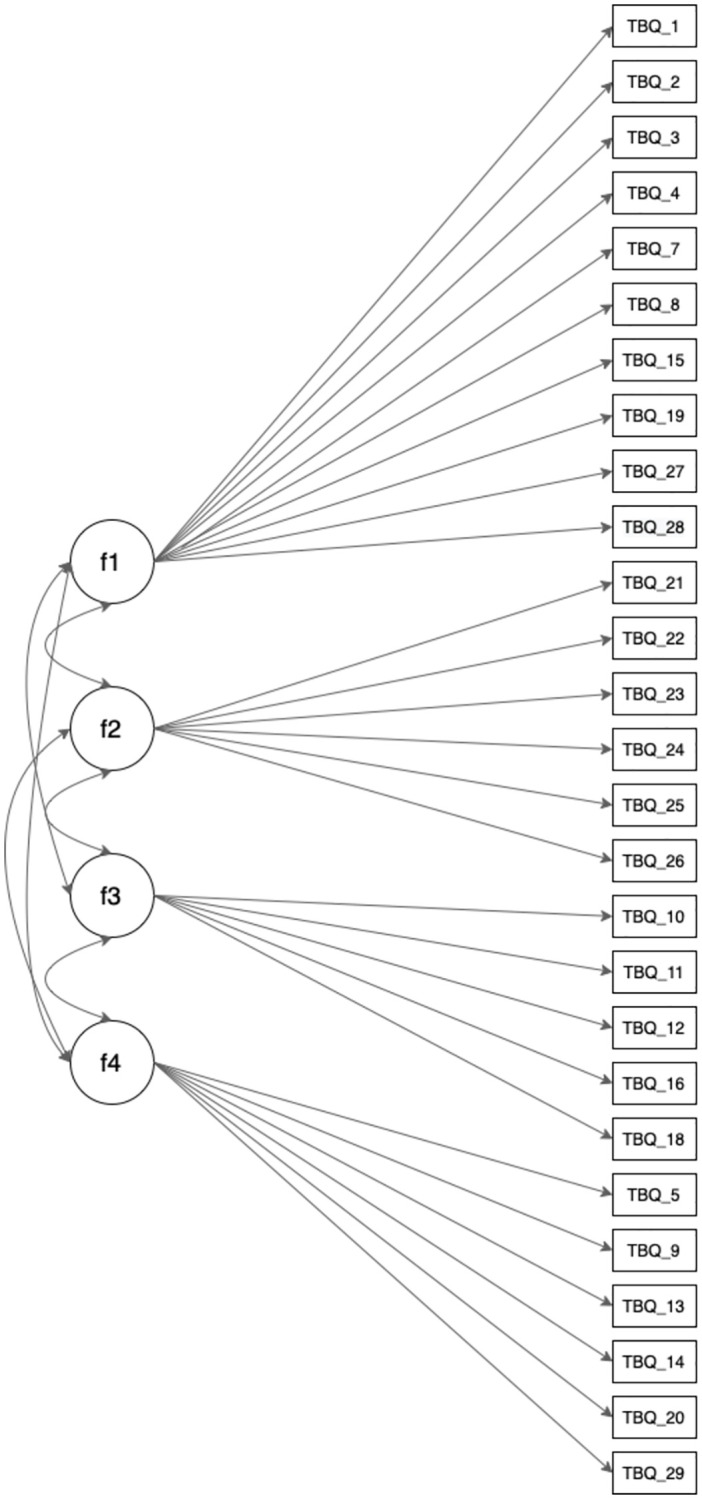
Graphical representation of the four-factor structure of the Tactile Biography Questionnaire (TBQ).

A graphical representation of the four-factor structure of the TBQ is provided in Fig 1.

As graphically reported in S7a-S7c Fig, no influential cases were detected.

Furthermore, internal consistency suggested excellent indices of reliability for all the four subscales (see Table 3).

**Table 3:** Reliability indices for the four factors.

	f1	f2	f3	f4
McDonald’s Omega	0.88	0.79	0.80	0.84

Childhood/Adolescent Touch Experience (f1), Comfort with Interpersonal Touch (f2), Fondness for Interpersonal Touch (f3) and Adult Touch Experience (f4) in the Calibration sample.

### Second step: Validation sample

A graphical representation of percentage distributions of items’ scores in the validation sample (*N*_*v*_ = 749) is displayed in the S4 and S5 Figs. Most of the items exhibit a skewed distribution. The results of the CFA revealed high and homogenous item loadings (S2 Table). Model fit indices obtained in the validation sample are summarized in Table 2.

### Measurement invariance across gender

Results of invariance analysis are reported, following the scheme suggested by Bowen and Masa [37], in Table 4. The first two models represent the four-factor TBQ model estimated separately in males and females. Results demonstrated that the model fit was adequate in both groups. Then, configural invariance was tested establishing a baseline model with all parameters freely estimated (unconstrained) across gender (*M*^M^ = *M*^F^). Fit indices showed that this model had adequate fit for the data suggesting that the factor structure is similar across groups. Subsequently, items’ loadings were constrained to be equal between females and males (Λ ^M^ = Λ ^F^). Fit statistics showed that this model did not result in a significant degradation of fit compared to the configural model (ΔCFI = .001; ΔRMSEA = -.001). After that, thresholds were constrained to equality between the two groups ((Λ, τ)^M^ = (Λ, τ)^F^). Fit indexes showed that this model (compared to the previous one) did not result in a significant degradation of fit (ΔCFI = < .001; ΔRMSEA = .006). Lastly, residuals variances were constrained to be equal across the two groups ((Λ, τ, Θ)^M^ = (Λ, τ, Θ)^F^), and fit indexes showed that this model did not result in a significant change of fit (ΔCFI = < .001; ΔRMSEA = < .001).

**Table 4:** Fit statistics for invariance testing.

		n	NNFI	CFI	ΔCFI	RMSEA	ΔRMSEA
1a	M (males)	339	0.969	0.972		0.098	
1b	F (females)	783	0.965	0.968		0.104	
2	*M*^M^ = *M*^F^	1122	0.967	0.969		0.102	
3	Λ ^M^ = Λ ^F^	1122	0.966	0.968	0.001	0.103	-0.001
4	(Λ, τ)^M^ = (Λ, τ)^F^	1122	0.969	0.968	<0.001	0.097	0.006
5	(Λ, τ, Θ)^M^ = (Λ, τ, Θ)^F^	1122	0.969	0.968	<0.001	0.097	<0.001

1a and 1b = estimated models for males and females separately; 2 = Configural invariance; 3 = Loadings invariance; 4 = Threshold invariance; 5 = Residual invariance.

CFI = comparative fit index; NNFI = Tucker–Lewis index; RMSEA = root mean square error of approximation.

Regarding measurement invariance evaluated with the bootstrap approach, we estimated the empirical distribution of the bootstrapped standardized loadings and calculated the overlapping area for each pair of items (S8 Fig). Results suggested that the construct was substantially invariant for all items except #1, 8, 9, 12, 15, 19, 20, 27, 28, 29, which overlapped for less than 30% between males and females.

### Concurrent validity

**Fig 2:**
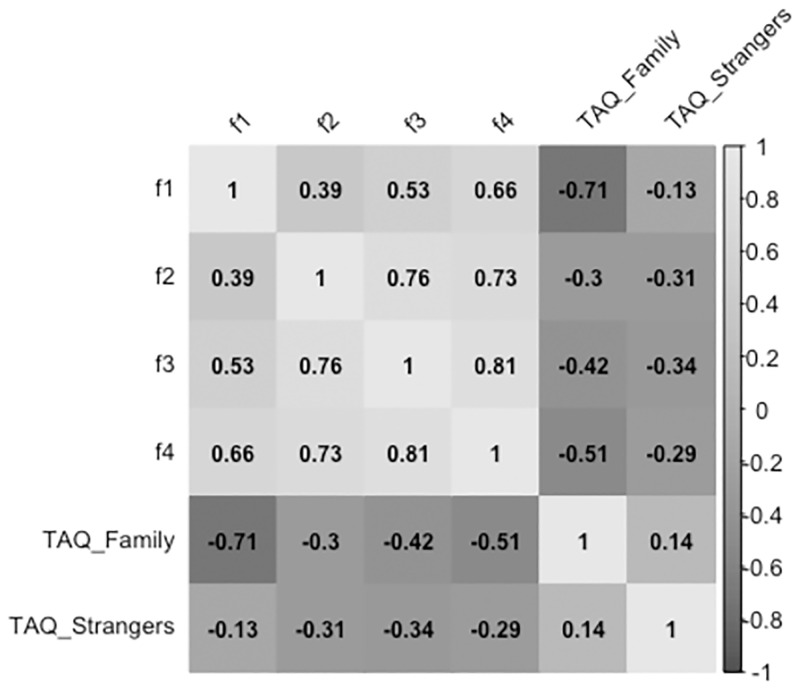
Bivariate correlation coefficients between family and stranger subscales of TAQ and factor scores of TBQ (n = 2040). Note. r, Pearson’s bivariate correlation coefficient and significance levels are reported; f1 = Childhood/Adolescent Touch Experience; f2 = Comfort with Interpersonal Touch; f3 = Fondness for Interpersonal Touch; f4 = Adult Touch Experience.

The concurrent validity of TBQ factors is summarized in Fig 2. TBQ factor scores negatively correlate with the included measures of touch avoidance (i.e., *Family* and *Stranger* subscales of TAQ).

### COVID-19 variables

Considering the examination of possible effects of the *COVID-19* pandemic on TBQ factor scores, no significant association emerged (S9 Fig).

## Discussion

In the present study we examined the factorial structure and the psychometric properties of the TBQ for use in the Italian population. A cross-validation strategy was used to investigate the validity of the four-factor structure of the TBQ presented by Beltran and colleagues [29]. In the first step (calibration sample), the four-factorial structure provided a good fit to the data, pointing out that the original structure proposed by the authors seems reliable for the Italian population as well. Sensitivity analysis was conducted and no influential cases were detected. This is informative of the low level of uncertainty of model estimates under different subsets of the data. Moreover, McDonald’s Omega highlighted good and excellent internal consistency coefficients of the four factors. Also in the second step, repeating the CFA on the validation sample, we obtained the same results as in the first step (calibration sample) confirming the validity of four-factorial structure on the TBQ in the Italian sample included in this study. Furthermore, the present study provided other measurement properties not investigated in the original analysis by Beltran and colleagues [29] which further suggest a remarkable usability of the questionnaire. In this regard, measurement invariance analysis between participant’s gender (males *vs*. females) were tested and established by hierarchical multi-group CFAs and bootstrap approaches. A recent meta-analysis highlighted differences between males and females in the perception of affective touch suggesting that females seem to be more sensitive to affective touch as well as to discriminative aspects of touch [30]. Moreover, compared to males, females appear to experience more physical touch throughout their lifespan and this may result in a different interpretation of the scale [47]. This issue has not been addressed in previous evaluations of the scale and is important as it shows that the TBQ scale scores are not confounded by gender and that they can be used to make meaningful comparisons between levels of males’ and females’ touch attitude. It should be noted that, using the bootstrap approach, even if the construct (i.e., affective experience of touch) was substantially invariant, items #1, 8, 9, 12, 15, 19, 20, 27, 28, 29 overlapped for less than 30% between males and females. Interestingly, 6 out of 10 (i.e., items #1, 8, 15, 19, 27, 28) are part of the *Childhood/Adolescent Touch Experience* factor. This seems to suggest that males and females interpret, at least partially, items related to early experiences of affective touch differently. Thus, testing measurement invariance also according to age would provide additional relevant properties of the scale. Therefore, we defer to future studies to investigate this aspect.

Regarding concurrent validity, present findings highlighted good to strong negative correlation coefficients between TBQ factors and TAQ subscales. The only unsatisfactory coefficient regards the correlation between *Childhood/Adolescent Touch Experience* and the *Strangers* subscale of the TAQ (*r = -*.*13*), which suggests that the amount of affective tactile experienced during childhood does not have a strong relationship with the amount of touch avoidance towards strangers. In other words, our result seems to suggest a fair independence of these two variables, which is a bit surprising given that it is not in line with the existing literature [33, 48]. The discrepancy could be, at least partially, related to the different kinds of tools and methodologies used in the current study with respect to the previous ones. Additional research is warranted to further explore the association between affective tactile experienced during childhood and touch avoidance towards strangers.

Finally, no significant associations were found between *COVID-19* related variables and TBQ factor scores. The social distancing regulations related to the *COVID-19* pandemic have reduced the ability to engage in interpersonal touch and this may alter the way in which affective touch is perceived [49]. A possible interpretation is that subjects’ pandemic-related experiences do not appear to have affected individual perceptions across the lifespan, suggesting that TBQ measures a relatively stable perception of affective touch experiences held by the individuals.

The present study is not without limitations. First, our sample was recruited by convenience sampling. This method may prevent the generalization of the present findings. Second, the included sample is not homogeneous in age as most of participants aged between 18 and 35 years. Future studies are encouraged to involve a more representative sample of the Italian population. Finally, the present work did not involve subjects from a clinical population with a diagnosis of psychopathology (e.g., depression, anxiety, anorexia nervosa). Exploring neurophysiological underpinnings of affective touch is becoming increasingly important in broadening the knowledge and improving a multidisciplinary approach to several mental-health issues such as anorexia nervosa, border personality and autism spectrum disorders [50–53]. Therefore, further studies of psychometric properties of the TBQ should be targeted also at the clinical population.

The Italian TBQ is a psychometrically sound and contextually sensitive measure of affective touch experiences that can be used to inform theory and research in this field. Overall, the findings of the present study showed that the Italian TBQ can become an interesting tool for education and psychology researchers to analyze the multidimensional nature of affective touch experiences across life in Italian population.

## Supporting information

S1 Fig Target model. The present study aimed at investigate the original four-factors structure of the Tactile Biography Questionnaire (TBQ) from 2097 Italian subjects (Females = 1342, 64%). A graphical representation of the original factors structure is reported below (S1 Fig).
(DOCX)

S2 Fig Comparing the original sample (*n* = 2612) and the current sample (*n* = 2040). Participants inclusion criteria were be more than 18 years old and be an Italian native speaker. The survey was filled by 2612 individuals (F = 1478). Participants with data missing from more than 25% of items were eliminated from subsequent analyses. Similarly, participants who agreed to participate and completed the 85% of items but did not fulfil the eligibility criteria were excluded from the final sample. From the initial sample, n = 572 subjects were excluded. In order to explore if excluded and included subjects were comparable, we graphically explored the frequencies of gender, answers to yes/no questions about COVID-19 (1. “have you ever tested positive for COVID-19?”, 2. “have some of your relatives ever tested positive for COVID-19?”, 3. “have you lost someone close to you because of COVID-19?) and the density of age and fear of COVID-19 (from 0 to 10) in the two groups. The two groups were comparable in terms of the variables considered.
(DOCX)

S3 Fig Comparison between the Calibration (n = 1246) and the Validation (n = 794) sample. In order to explore psychometric properties and factor-structure of the TBQ, a cross validation with a two-step analytic approach was carried out. The original sample was split into two independent randomly chosen sub-samples, the calibration sample, which included Nc = 1246 subjects, and the validation sample, which included Nv = 794 subjects. Here we report the graphical representation of the frequencies of gender and answers to yes/no questions about COVID-19 (1. “have you ever tested positive for COVID-19?”, 2. “have some of your relatives ever tested positive for COVID-19?”, 3. “have you lost someone close to you because of COVID-19?) as well as the density of age and fear of COVID-19 in the Calibration and Validation sample.
(DOCX)

S4 Fig Percentage distribution of items’ scores in the Calibration (n = 1246) and Validation (n = 794) sample. Items’ response values and percentages are reported on the x and y axis respectively. Item 30 it is not represented in the figure as it is a multiple-choice item.
(DOCX)

S5 Fig Frequencies distribution of items’ scores in the Calibration (n = 1246) and Validation (n = 794) sample. Frequencies distribution of items’ scores in the Calibration (n = 1246) and Validation (n = 794) sample. Items’ response values and frequencies are reported on the x and y axis respectively. Item 30 it is not represented in the figure as it is a multiple-choice item.
(DOCX)

S6 Fig Frequency (and percentage) of missing values for each item of the TBQ. The calibration sample is composed by 1246 subjects. In S6 Fig are represented the frequencies and percentages of missing values for each item of the TBQ. Given the low percentage of missing data, we decided to report in the paper only results derived from the listwise deletion strategy, excluding subjects with missing values (N = 78) from the analyses. In S3a and S3b Table (pag. 12–13), we reported results obtained by using the full information maximum likelihood approach for imputing missing data.
(DOCX)

S7 Fig **a.** Likelihood Distance (*LD)* for each observation in the Calibration sample. Each point represent the LD when the observation is deleted from the sample. Here we evaluate case influence which refers to the impact of a case on study results quantified by detection statistics. This approach compares the solutions obtained from the original sample with those obtained from the sample excluding case i, where i represents each case in turn. A way to evaluate case influence in SEM is the Likelihood Distance (Ldi) (1). Specifically, it evaluates the influence of a case on the global fit of the model. The higher value of LDi, the greater is the influence. the global fit of the model. In the present study LDi was evaluated with respect to the TBQ four-factors model tested in the Calibration data sample (first step). The graph below highlights the absence of cases with a significant influence on the global fit of the model. **b.** The *ΔCFI* difference (*ΔCFI*) for each observation in the Calibration sample. We also evaluated the influence of each case on the global fit of the model computing the CFI difference (ΔCFI). This measure highlight the magnitude and the direction of influence. Thus, positive values of ΔCFI indicate that by removing case i the model is improved while negative values indicate the opposite. As seen in the graph below, no influential cases were detected. Again, each point represent the ΔCFI when the observation is deleted from the sample. **c.** The Generalized Cook’s Distance for each observation in the Calibration sample. Each point represent the Generalized Cook’s Distance when the observation is deleted from the sample. We used Generalized Cook’s Distance in order to evaluate the influence of a case on parameter estimates of our model. As seen from the graph below, by removing case i parameter estimates did not change significantly.
(ZIP)

S8 Fig Standardized factor loading bootstrap distributions of the TBQ items in Males and Females. This approach allows to estimate the sampling distribution of a statistic of interest (e.g., factor loadings) by resampling with replacement from the original sample without normality assumption. We created 5000 bootstrapped replicates sampling from the original data in order to ensure that the actual number of acceptable solutions (n = 3279) was sufficiently large, extracted the empirical distribution of each factor loading, and evaluated the overlapping area for each corresponding pair of items (e.g., item 1 for males and item 1 for females). Again, f1 = Childhood/Adolescent Touch Experience; f2 = Comfort with Interpersonal Touch; f3 = Fondness for Interpersonal Touch; f4 = Adult Touch Experience.
(DOCX)

S9 Fig Pearson’s bivariate correlation coefficients between TBQ factors scores and COVID-19 related variables. As the data collection was performed during COVID-19 pandemic, a specific section of the survey was devoted to collect information about the impact of COVID-19 situation on participants and relatives using the following yes/no questions: 1. “have you ever tested positive for COVID-19?”, 2. “have some of your relatives ever tested positive for COVID-19?”, 3. “have you lost someone close to you because of COVID-19?”. The last question, 4. “How scared are you of COVID-19?”, could be answered with a rating scale ranging from 0 to 10. No significant association between TBQ factor scores and COVID-19 related variable emerged.
(DOCX)

S1 Table Italian and English version of the Tactile Biography Questionnaire (TBQ). We translated the TBQ from English to Italian; subsequently, a translator practiced in both languages proceeded with the back translation; finally, we asked the authors of the instrument if the back translation was adequate. The Italian version of the TBQ is reported below beside the original version. (R) indicates reversed item.
(DOCX)

S2 Table Factor loadings in the Calibration (n = 1246) and Validation (n = 749) sample. Factor loadings in the Calibration (n = 1246) and Validation (n = 749) sample. f1 = Childhood/Adolescent Touch Experience; f2 = Comfort with Interpersonal Touch; f3 = Fondness for Interpersonal Touch; f4 = Adult Touch Experience.
(DOCX)

S3 Table **a.** Fit indices obtained by imputing missing data with full information maximum likelihood approach (film) in the Calibration Sample. CFI = comparative fit index; NNFI = Tucker–Lewis index; RMSEA = root mean square error of approximation; SRMR = Standardized root mean square residual. TCD = total coefficient of determination. **b.** Factor loadings obtained with data imputation in the Calibration Sample. f1 = Childhood/Adolescent Touch Experience; f2 = Comfort with Interpersonal Touch; f3 = Fondness for Interpersonal Touch; f4 = Adult Touch Experience.
(ZIP)
