# Supplementary material for: Tactile Biography Questionnaire: A contribution to its validation in an Italian sample
Source: PLoS One. 2022 Sep 15;17(9):e0274477. doi: 10.1371/journal.pone.0274477 (PMC9477375; doi:10.1371/journal.pone.0274477)

**S1 Fig.** **Target Model**.

The present study aimed at investigate the original four-factors structure of the Tactile Biography Questionnaire (TBQ) from 2097 Italian subjects (Females = 1342, 64%). A graphical representation of the original factors structure is reported below.


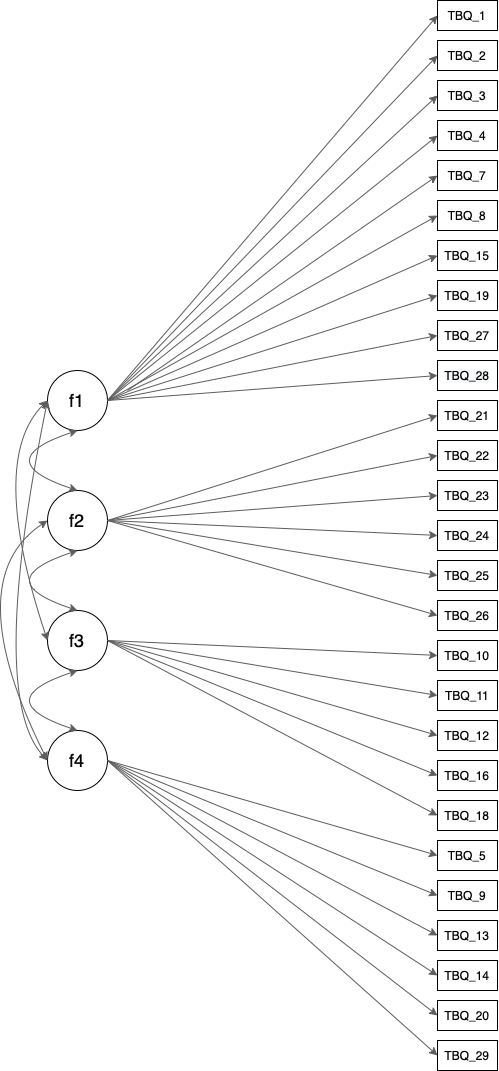

Supplement: S1 Fig — The present study aimed at investigate the original four-factors structure of the Tactile Biography Questionnaire (TBQ) from 2097 Italian subjects (Females = 1342, 64%). A graphical representation of the original factors structure is reported below (S1 Fig). (DOCX) [file pone.0274477.s001.docx]
