# Supplementary material for: Tactile Biography Questionnaire: A contribution to its validation in an Italian sample
Source: PLoS One. 2022 Sep 15;17(9):e0274477. doi: 10.1371/journal.pone.0274477 (PMC9477375; doi:10.1371/journal.pone.0274477)

**S2 Fig. Comparing the original sample (*n* = 2612) and the current sample (*n* = 2040).**

Participants inclusion criteria were be more than 18 years old and be an Italian native speaker. The survey was filled by 2612 individuals (F = 1478). Participants with data missing from more than 25% of items were eliminated from subsequent analyses. Similarly, participants who agreed to participate and completed the 85% of items but did not fulfil the eligibility criteria were excluded from the final sample. From the initial sample, *n* = 572 subjects were excluded. In order to explore if excluded and included subjects were comparable, we graphically explored the frequencies of gender, answers to yes/no questions about COVID-19 (1. “have you ever tested positive for COVID-19?”, 2. “have some of your relatives ever tested positive for COVID-19?”, 3. “have you lost someone close to you because of COVID-19?) and the density of age and fear of COVID-19 (from 0 to 10) in the two groups. The two groups were comparable in terms of the variables considered.


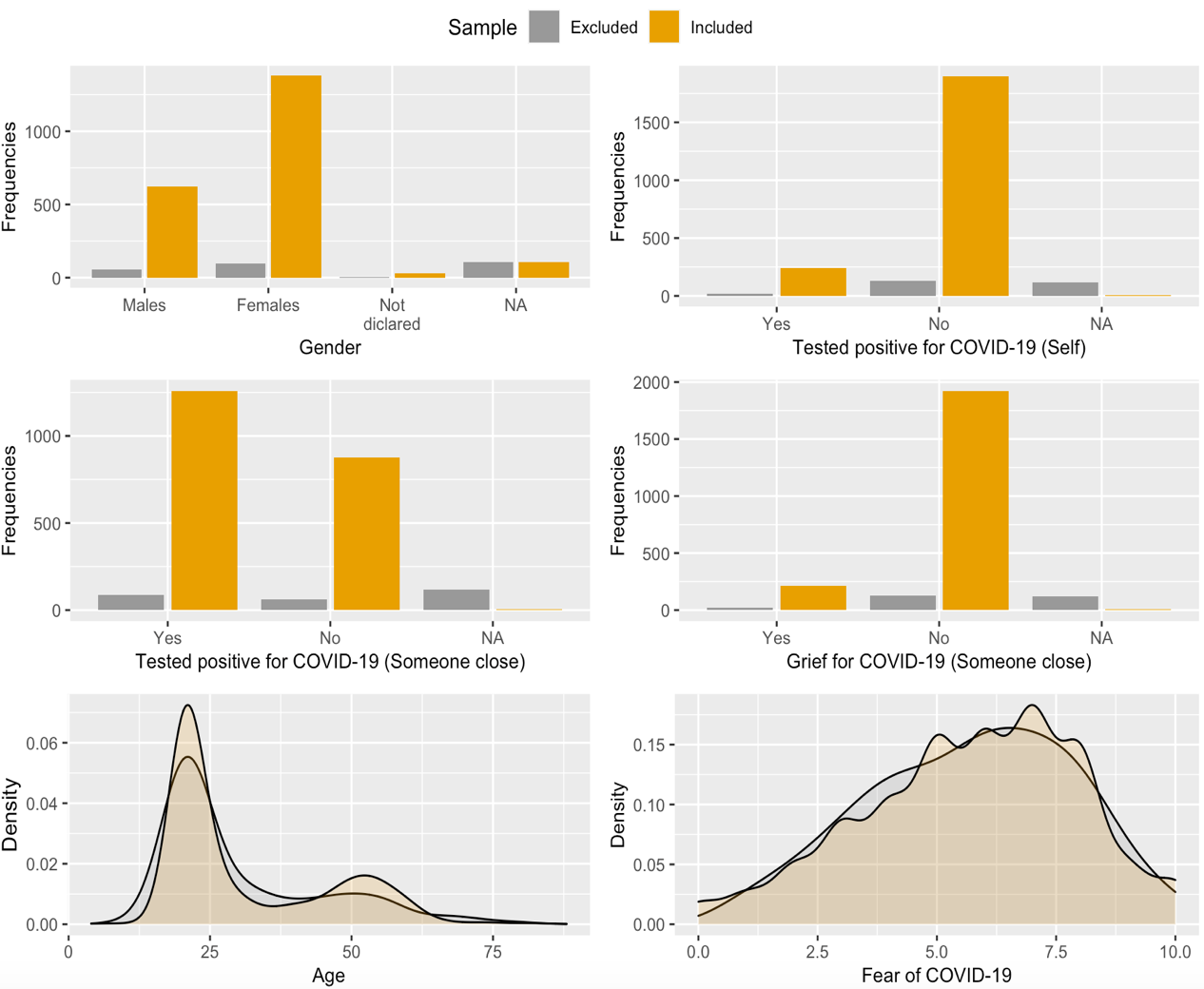

Supplement: S2 Fig — Participants inclusion criteria were be more than 18 years old and be an Italian native speaker. The survey was filled by 2612 individuals (F = 1478). Participants with data missing from more than 25% of items were eliminated from subsequent analyses. Similarly, participants who agreed to participate and completed the 85% of items but did not fulfil the eligibility criteria were excluded from the final sample. From the initial sample, n = 572 subjects were excluded. In order to explore if excluded and included subjects were comparable, we graphically explored the frequencies of gender, answers to yes/no questions about COVID-19 (1. “have you ever tested positive for COVID-19?”, 2. “have some of your relatives ever tested positive for COVID-19?”, 3. “have you lost someone close to you because of COVID-19?) and the density of age and fear of COVID-19 (from 0 to 10) in the two groups. The two groups were comparable in terms of the variables considered. (DOCX) [file pone.0274477.s002.docx]
