# Supplementary material for: Tactile Biography Questionnaire: A contribution to its validation in an Italian sample
Source: PLoS One. 2022 Sep 15;17(9):e0274477. doi: 10.1371/journal.pone.0274477 (PMC9477375; doi:10.1371/journal.pone.0274477)

**S3 Fig. Comparing the Calibration (n = 1246) and the Validation (n = 794) sample.**

In order to explore psychometric properties and factor-structure of the TBQ, a cross validation with a two-step analytic approach was carried out. The original sample was split into two independent randomly chosen sub-samples, the calibration sample, which included N_c_ = 1246 subjects, and the validation sample, which included N_v_ = 794 subjects. Here we report the graphical representation of the frequencies of gender and answers to yes/no questions about COVID-19 (1. “have you ever tested positive for COVID-19?”, 2. “have some of your relatives ever tested positive for COVID-19?”, 3. “have you lost someone close to you because of COVID-19?) as well as the density of age and fear of COVID-19 in the Calibration and Validation sample.


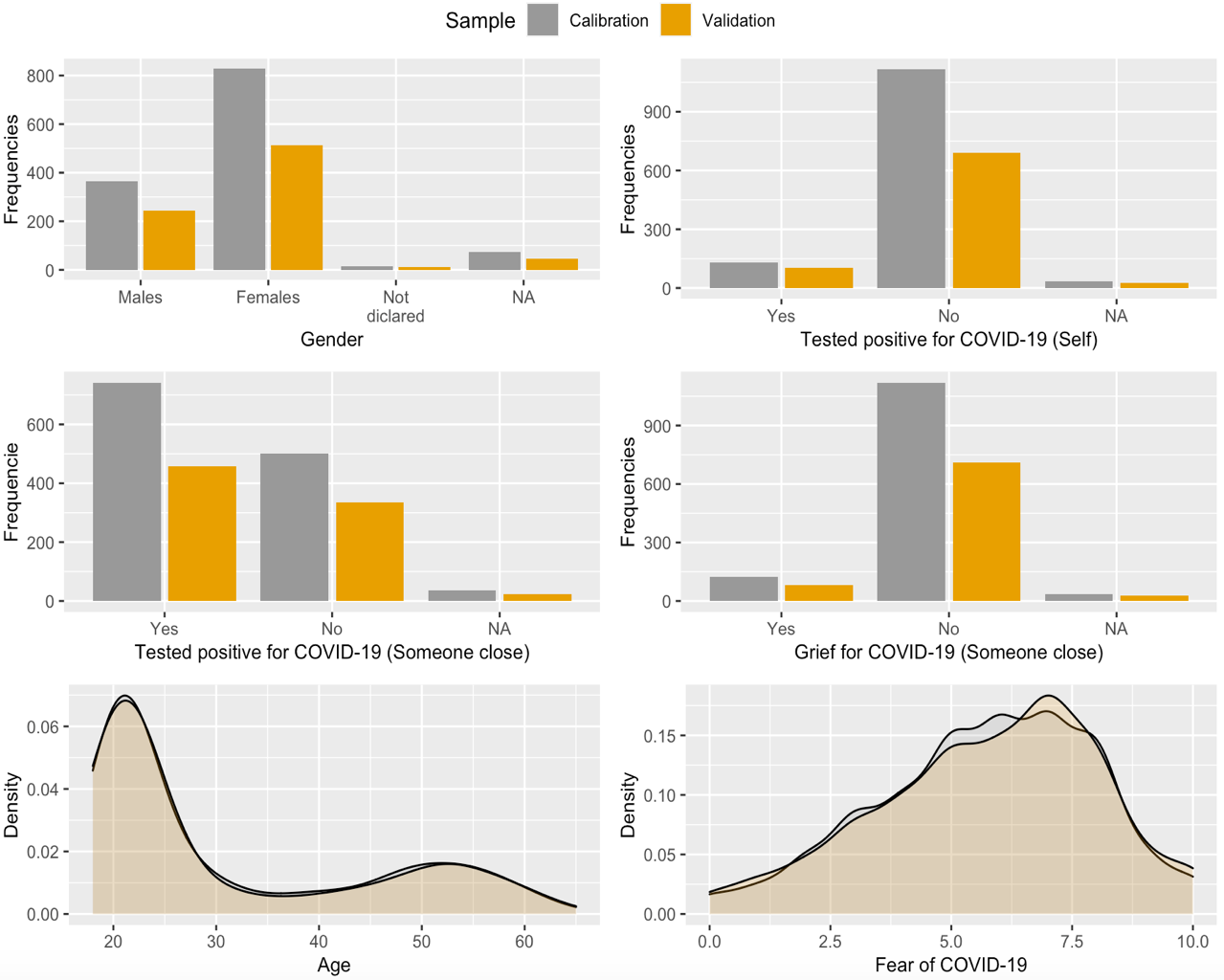

Supplement: S3 Fig — In order to explore psychometric properties and factor-structure of the TBQ, a cross validation with a two-step analytic approach was carried out. The original sample was split into two independent randomly chosen sub-samples, the calibration sample, which included Nc = 1246 subjects, and the validation sample, which included Nv = 794 subjects. Here we report the graphical representation of the frequencies of gender and answers to yes/no questions about COVID-19 (1. “have you ever tested positive for COVID-19?”, 2. “have some of your relatives ever tested positive for COVID-19?”, 3. “have you lost someone close to you because of COVID-19?) as well as the density of age and fear of COVID-19 in the Calibration and Validation sample. (DOCX) [file pone.0274477.s003.docx]
