# Supplementary material for: Tactile Biography Questionnaire: A contribution to its validation in an Italian sample
Source: PLoS One. 2022 Sep 15;17(9):e0274477. doi: 10.1371/journal.pone.0274477 (PMC9477375; doi:10.1371/journal.pone.0274477)

**S4 Fig. Percentage distributions of items’ scores in the Calibration (n = 1246) and Validation (n = 749) sample.**

Items’ response values and percentages are reported on the x and y axis respectively. Item 30 it is not represented in the figure as it is a multiple-choice item.


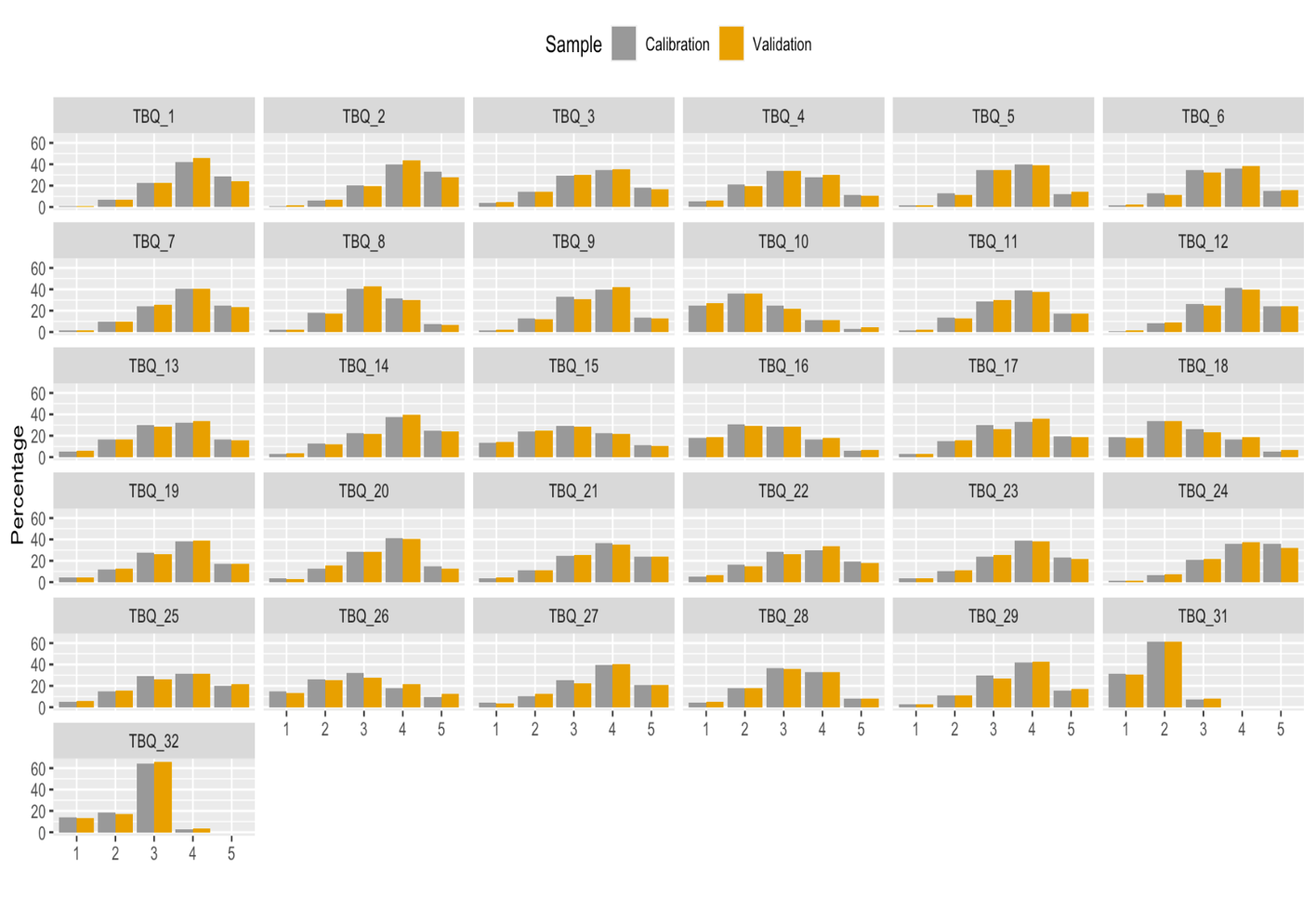

Supplement: S4 Fig — Items’ response values and percentages are reported on the x and y axis respectively. Item 30 it is not represented in the figure as it is a multiple-choice item. (DOCX) [file pone.0274477.s004.docx]
