# Supplementary material for: Tactile Biography Questionnaire: A contribution to its validation in an Italian sample
Source: PLoS One. 2022 Sep 15;17(9):e0274477. doi: 10.1371/journal.pone.0274477 (PMC9477375; doi:10.1371/journal.pone.0274477)

**S6 Fig. Frequency (and percentage) of missing values for each item of the TBQ.**

The calibration sample is composed by 1246 subjects. In figure S6 are represented the frequencies and percentages of missing values for each item of the TBQ. Given the low percentage of missing data, we decided to report in the paper only results derived from the listwise deletion strategy, excluding subjects with missing values (N = 78) from the analyses. In table S3a and S3b (pag. 12-13) of the Supplementary Materials we reported results obtained by using the full information maximum likelihood approach for imputing missing data.


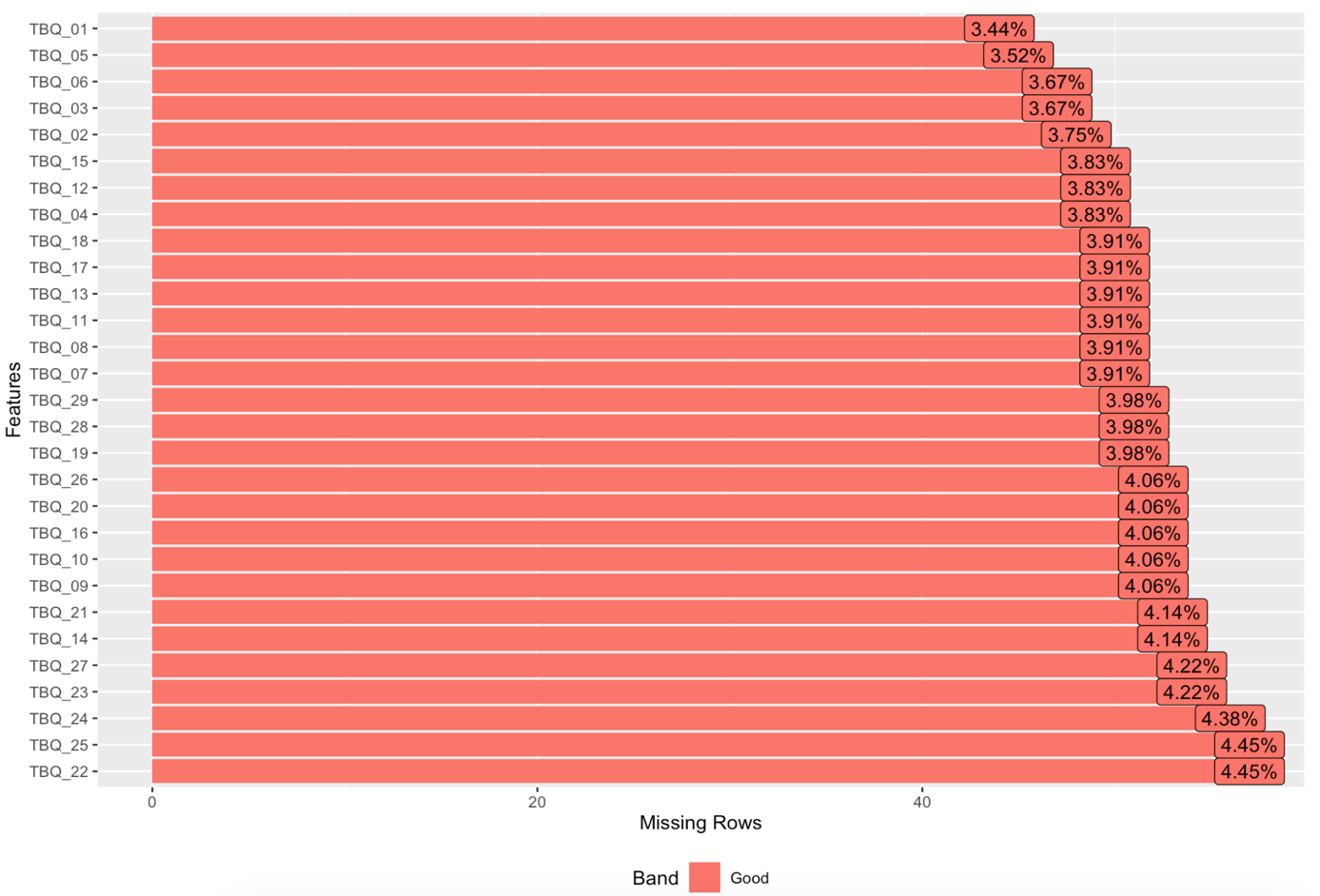

Supplement: S6 Fig — The calibration sample is composed by 1246 subjects. In S6 Fig are represented the frequencies and percentages of missing values for each item of the TBQ. Given the low percentage of missing data, we decided to report in the paper only results derived from the listwise deletion strategy, excluding subjects with missing values (N = 78) from the analyses. In S3a and S3b Table (pag. 12–13), we reported results obtained by using the full information maximum likelihood approach for imputing missing data. (DOCX) [file pone.0274477.s006.docx]
