# Supplementary material for: Tactile Biography Questionnaire: A contribution to its validation in an Italian sample
Source: PLoS One. 2022 Sep 15;17(9):e0274477. doi: 10.1371/journal.pone.0274477 (PMC9477375; doi:10.1371/journal.pone.0274477)

**S7c Fig. The Generalized Cook’s Distance for each observation in the Calibration sample.**

Each point represents the Generalized Cook’s Distance when the observation is deleted from the sample. We used Generalized Cook’s Distance in order to evaluate the influence of a case on parameter estimates of our model. As seen from the graph below, by removing case *i* parameter estimates did not change significantly.


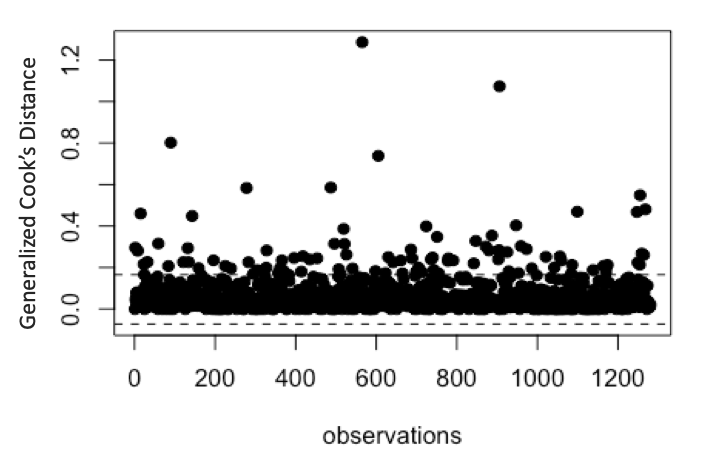

Supplement: S7 Fig — a. Likelihood Distance (LD) for each observation in the Calibration sample. Each point represent the LD when the observation is deleted from the sample. Here we evaluate case influence which refers to the impact of a case on study results quantified by detection statistics. This approach compares the solutions obtained from the original sample with those obtained from the sample excluding case i, where i represents each case in turn. A way to evaluate case influence in SEM is the Likelihood Distance (Ldi) (1). Specifically, it evaluates the influence of a case on the global fit of the model. The higher value of LDi, the greater is the influence. the global fit of the model. In the present study LDi was evaluated with respect to the TBQ four-factors model tested in the Calibration data sample (first step). The graph below highlights the absence of cases with a significant influence on the global fit of the model. b. The ΔCFI difference (ΔCFI) for each observation in the Calibration sample. We also evaluated the influence of each case on the global fit of the model computing the CFI difference (ΔCFI). This measure highlight the magnitude and the direction of influence. Thus, positive values of ΔCFI indicate that by removing case i the model is improved while negative values indicate the opposite. As seen in the graph below, no influential cases were detected. Again, each point represent the ΔCFI when the observation is deleted from the sample. c. The Generalized Cook’s Distance for each observation in the Calibration sample. Each point represent the Generalized Cook’s Distance when the observation is deleted from the sample. We used Generalized Cook’s Distance in order to evaluate the influence of a case on parameter estimates of our model. As seen from the graph below, by removing case i parameter estimates did not change significantly. (ZIP) [file pone.0274477.s007.zip › S7c_Fig.docx]
