# Supplementary material for: Tactile Biography Questionnaire: A contribution to its validation in an Italian sample
Source: PLoS One. 2022 Sep 15;17(9):e0274477. doi: 10.1371/journal.pone.0274477 (PMC9477375; doi:10.1371/journal.pone.0274477)

**S8 Fig. Standardized factor loading bootstrap distributions of the TBQ items in Males and Females.**

This approach allows to estimate the sampling distribution of a statistic of interest (e.g., factor loadings) by resampling with replacement from the original sample without normality assumption. We created 5000 bootstrapped replicates sampling from the original data in order to ensure that the actual number of acceptable solutions (*n* = 3279) was sufficiently large, extracted the empirical distribution of each factor loading, and evaluated the overlapping area for each corresponding pair of items (e.g., item 1 for males and item 1 for females). Again, f1= Childhood/Adolescent Touch Experience; f2 = Comfort with Interpersonal Touch; f3 = Fondness for Interpersonal Touch; f4 = Adult Touch Experience.


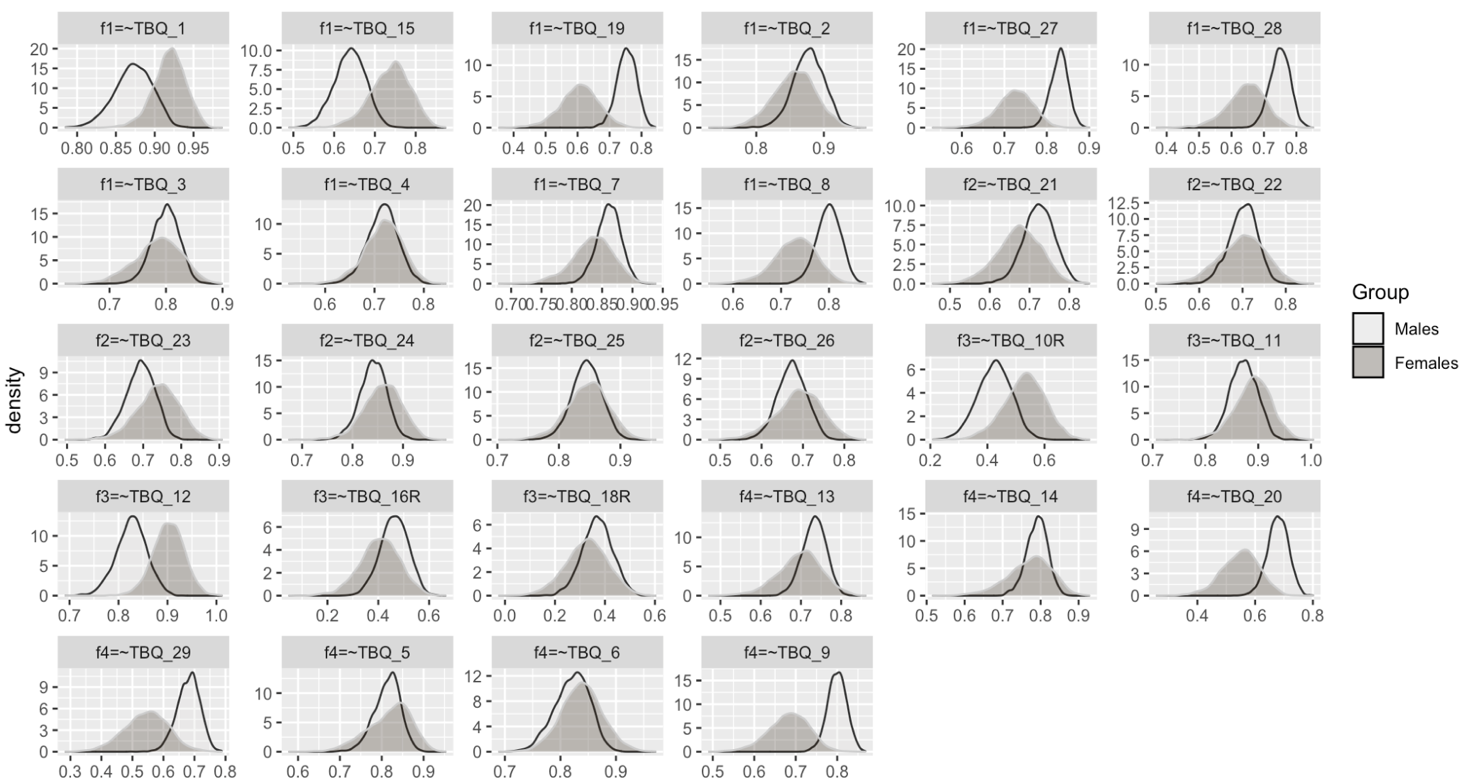

Supplement: S8 Fig — This approach allows to estimate the sampling distribution of a statistic of interest (e.g., factor loadings) by resampling with replacement from the original sample without normality assumption. We created 5000 bootstrapped replicates sampling from the original data in order to ensure that the actual number of acceptable solutions (n = 3279) was sufficiently large, extracted the empirical distribution of each factor loading, and evaluated the overlapping area for each corresponding pair of items (e.g., item 1 for males and item 1 for females). Again, f1 = Childhood/Adolescent Touch Experience; f2 = Comfort with Interpersonal Touch; f3 = Fondness for Interpersonal Touch; f4 = Adult Touch Experience. (DOCX) [file pone.0274477.s008.docx]
