# Supplementary material for: Tactile Biography Questionnaire: A contribution to its validation in an Italian sample
Source: PLoS One. 2022 Sep 15;17(9):e0274477. doi: 10.1371/journal.pone.0274477 (PMC9477375; doi:10.1371/journal.pone.0274477)

**S9 Fig. Pearson’s bivariate correlation coefficients between TBQ factors scores and COVID-19 related variables.**

As the data collection was performed during COVID-19 pandemic, a specific section of the survey was devoted to collect information about the impact of COVID-19 situation on participants and relatives using the following yes/no questions: 1. “have you ever tested positive for COVID-19?”, 2. “have some of your relatives ever tested positive for COVID-19?”, 3. “have you lost someone close to you because of COVID-19?”. The last question, 4. "How scared are you of COVID-19?", could be answered with a rating scale ranging from 0 to 10. No significant association between TBQ factor scores and COVID-19 related variable emerged.


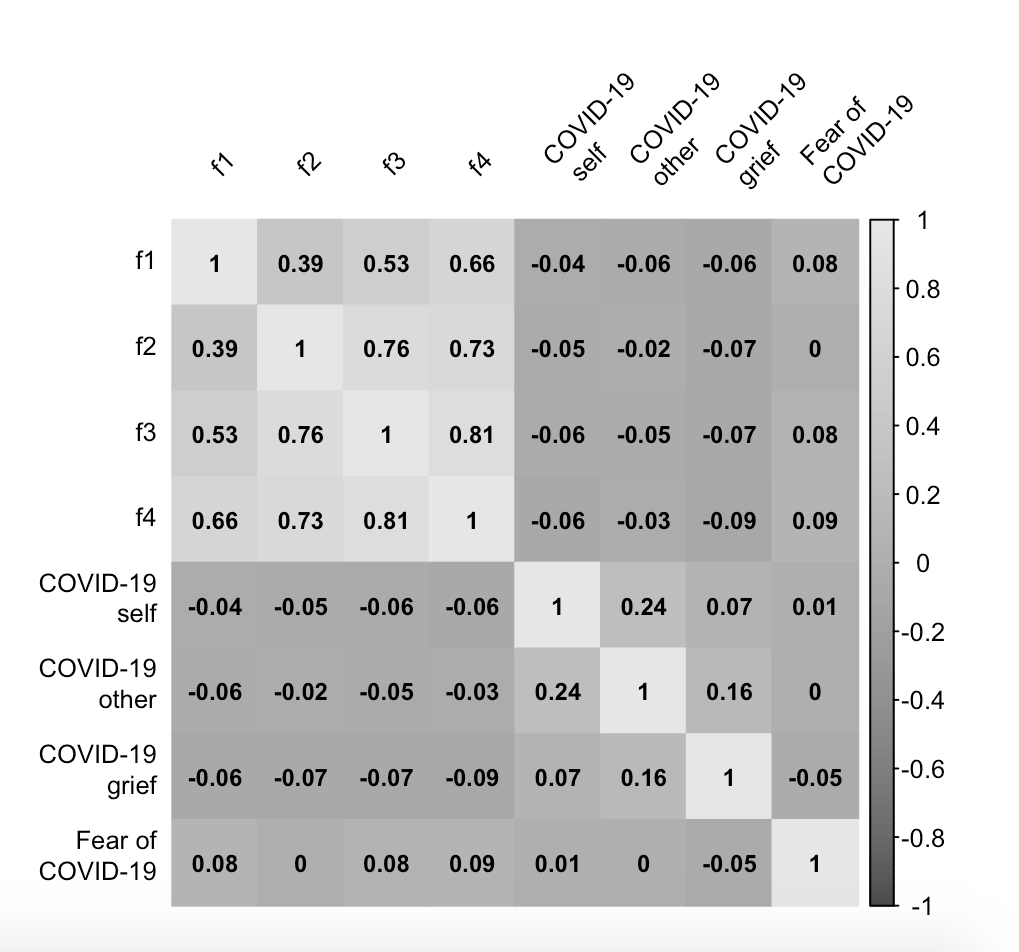

Supplement: S9 Fig — As the data collection was performed during COVID-19 pandemic, a specific section of the survey was devoted to collect information about the impact of COVID-19 situation on participants and relatives using the following yes/no questions: 1. “have you ever tested positive for COVID-19?”, 2. “have some of your relatives ever tested positive for COVID-19?”, 3. “have you lost someone close to you because of COVID-19?”. The last question, 4. “How scared are you of COVID-19?”, could be answered with a rating scale ranging from 0 to 10. No significant association between TBQ factor scores and COVID-19 related variable emerged. (DOCX) [file pone.0274477.s009.docx]
