# Supplementary material for: Tactile Biography Questionnaire: A contribution to its validation in an Italian sample
Source: PLoS One. 2022 Sep 15;17(9):e0274477. doi: 10.1371/journal.pone.0274477 (PMC9477375; doi:10.1371/journal.pone.0274477)
Supplement: S1 Table — We translated the TBQ from English to Italian; subsequently, a translator practiced in both languages proceeded with the back translation; finally, we asked the authors of the instrument if the back translation was adequate. The Italian version of the TBQ is reported below beside the original version. (R) indicates reversed item. (DOCX) [file pone.0274477.s010.docx]

**S1 Table.** **Translation of the original version of the Tactile Biography Questionnaire (TBQ)**.

We translated the TBQ from English to Italian; subsequently, a translator practiced in both languages proceeded with the back translation; finally, we asked the authors of the instrument if the back translation was adequate. The Italian version of the TBQ is reported below beside the original version. (R) indicates reversed item.

| 1. | Da bambina/o ho ricevuto del tocco affettivo dai membri della famiglia (genitori/caregiver). | As a child I received affective touch from family members (parents/caregivers). |
| --- | --- | --- |
| 2. | Da bambina/o i miei genitori/caregiver hanno usato il contatto corporeo (per esempio: carezze, abbracci, ecc.) per confortarmi quando ero malata/o o afflitta/o. | As a child my parents/caregivers would use bodily contact (e.g.: caressing, hugging, etc) to comfort me when ill/distressed. |
| 3. | Da bambina/o i miei genitori/caregiver usavano il contatto corporeo (per esempio: carezze, abbracci, ecc.) per congratularsi con me/per darmi un feedback positivo. | As a child my parents/caregivers would use bodily contact (e.g.: caressing, hugging, etc) to congratulate me/ give me positive feedback. |
| 4. | Da bambina/o, ho ricevuto del tocco affettivo da amici/fratelli. | As a child, I received affective touch from friends/siblings. |
| 5. | Nella mia vita adulta ho ricevuto del tocco affettivo da amici stretti o membri della famiglia. | In my adult life I have received affective touch from close friends or family members |
| 6. | Nella mia vita adulta ho utilizzato un tocco affettivo con amici stretti o membri della famiglia. | In my adult life I have given affective touch to close friends or family members |
|  | Quanto spesso hai sperimentato il tocco affettivo in diversi momenti della vita? | Please indicate how frequently you experienced affective touch in these different life moments: |
| 7. | Infanzia | Childhood |
| 8. | Adolescenza | Adolescence |
| 9. | Età adulta | Adulthood |
| 10. | Da bambina/o, non mi piaceva essere abbracciata/o dai miei familiari o amici. (R) | As a child, I did not like to be hugged by my family members or friends. |
| 11. | Mi è sempre piaciuto ricevere carezze da qualcuno che mi è vicino. | I have always liked to receive caresses from someone that I am close to. |
| 12. | Mi è sempre piaciuto ricevere contatto corporeo confortante (per esempio un abbraccio) da qualcuno che mi è vicino quando sono in difficoltà. | I’ve always I liked to receive comforting bodily contact (e.g. hug) from someone I am close to when distressed |
| 13. | Ho sempre trovato facile confortare amici/familiari abbracciando o toccando la loro mano/braccio. | I’ve always I found it easy to comfort friends/family members by hugging or touching their hand/arm |
| 14. | Riconosco nella mia storia personale di aver utilizzato il tocco affettivo (per esempio: abbracci, carezze, tocco gentile nel braccio) come un modo per comunicare affetto. | I recognize in my personal history that I use affective touch (e.g.: hugs, caress, gentle touch in the arm) as a way to communicate affection. |
| 15. | Mentre crescevo, in situazioni di stress andavo dai miei genitori/caregiver in cerca di tocco affettivo (abbracci, coccole, carezze) | While growing up, upon stressful situations I would go to my parents/caregivers in search of affective touch (hugs, cuddling, caressing) |
| 16. | Durante la crescita rifiutavo il tocco affettivo (per esempio abbracci, carezze) dei miei genitori/accompagnatori. (R) | While growing up I would reject affective touch (e.g. hugs, caresses) from my parents/caregivers. |
|  |  |  |
| 17. | Riconosco nella mia storia personale il bisogno/desiderio di contatto fisico affettivo (abbracci, carezze, braccio intorno alla spalla) quando angosciata/o. | I recognize in my personal history the need/ desire of physical affective contact (hugs, caress, arm around shoulder) when I am distressed. |
| 18. | Riconosco nella mia storia personale il bisogno/desiderio di evitare il contatto fisico affettivo (abbracci, carezze, braccio intorno alla spalla) quando sono angosciata/o. (R) | I recognize in my personal history the need/ desire to avoid physical affective contact (hugs, caress, arm around shoulder) when I am distressed. |
| 19. | Sono soddisfatta/o della quantità di tocco affettivo che ho ricevuto durante la mia storia personale. | I am satisfied with the amount of affective touch I received throughout my personal story. |
| 20. | Sono soddisfatta/o della quantità di tocco affettivo che ho dato agli altri nel corso della mia storia personale. | I am satisfied with the amount of affective touch I gave to others throughout my personal story. |
|  | Quanto ti senti a tuo agio con questi tipi di tocco affettivo interpersonale nelle relazioni strette (romantiche e non)? | Please indicate how comfortable do you feel with these types of affective interpersonal touch in close (romantic and non-romantic) relationships |
| 21. | Tenersi per mano | Holding hands |
| 21. | Tenere la mano intorno alla spalla. | Hand around the shoulder |
| 23. | Toccare l'avambraccio o il braccio dell'altra persona per dare conforto. | Touch forearm or arm of other person to give comfort |
| 24. | Abbracciare | Hugging |
| 25. | Accarezzare | Caressing/stroking |
| 26. | Massaggiare | Massaging |
|  | Quanto sei soddisfatta/o della quantità di tocco affettivo che hai sperimentato nelle relazioni strette, in diversi momenti della tua vita? | How happy you are with the amount of affective touch you experienced in close relationships |
| 27. | Infanzia | Childhood |
| 28. | Adolescenza | Adolescence |
| 29. | Età adulta | Adulthood |
| 30. | Il tocco affettivo nelle relazioni interpersonali strette genera in me...(puoi selezionare più di una risposta): | Affective touch in close interpersonal relationships generates in me… (selecting more than 1 option is valid |
|  | Calma/rilassatezza | Calmness/relaxation |
|  | Imbarazzo | Embarrassmentt |
|  | Gioia | Enjoyment |
|  | Felicità | Happiness |
|  | Rifiuto | Rejection |
|  | Disgusto | Disgust |
|  | Conforto | Comfort |
|  | Irritazione | Irritation |
|  | Disagio | Discomfort |
|  | Piacere | Pleasure |
|  |  |  |
| 31. | Riconosco nella mia storia personale di aver vissuto esperienze negative/spiacevoli legate al tocco interpersonale | I recognize in my personal history the presence of negative/unpleasant experiences involving interpersonal |
|  | Sì | Yes |
|  | No | No |
|  | Preferisco non rispondere | Prefer not to answer |
|  |  |  |
| 32. | Nelle relazioni intime ho sempre preferito: | In close relationships have always preferred to: |
|  | dare contatto affettivo | give affective touch |
|  | ricevere contatto affettivo | receive affective touch |
|  | non ho preferenze (mi piacciono entrambe le cose) | does not matter (I like both the same) |
|  | non ho preferenze (non mi piace nessuna delle due cose) | does not matter (I dislike both the same) |
