# Supplementary material for: Tactile Biography Questionnaire: A contribution to its validation in an Italian sample
Source: PLoS One. 2022 Sep 15;17(9):e0274477. doi: 10.1371/journal.pone.0274477 (PMC9477375; doi:10.1371/journal.pone.0274477)
Supplement: S2 Table — Factor loadings in the Calibration (n = 1246) and Validation (n = 749) sample. f1 = Childhood/Adolescent Touch Experience; f2 = Comfort with Interpersonal Touch; f3 = Fondness for Interpersonal Touch; f4 = Adult Touch Experience. (DOCX) [file pone.0274477.s011.docx]

**S2 Table. Factor loadings in the Calibration (n = 1246) and Validation (n = 749) sample.**

Factor loadings in the Calibration (n = 1246) and Validation (n = 749) sample. f1= Childhood/Adolescent Touch Experience; f2 = Comfort with Interpersonal Touch; f3 = Fondness for Interpersonal Touch; f4 = Adult Touch Experience.
