# Supplementary material for: Tactile Biography Questionnaire: A contribution to its validation in an Italian sample
Source: PLoS One. 2022 Sep 15;17(9):e0274477. doi: 10.1371/journal.pone.0274477 (PMC9477375; doi:10.1371/journal.pone.0274477)
Supplement: S3 Table — a. Fit indices obtained by imputing missing data with full information maximum likelihood approach (film) in the Calibration Sample. CFI = comparative fit index; NNFI = Tucker–Lewis index; RMSEA = root mean square error of approximation; SRMR = Standardized root mean square residual. TCD = total coefficient of determination. b. Factor loadings obtained with data imputation in the Calibration Sample. f1 = Childhood/Adolescent Touch Experience; f2 = Comfort with Interpersonal Touch; f3 = Fondness for Interpersonal Touch; f4 = Adult Touch Experience. (ZIP) [file pone.0274477.s012.zip › S3b_Table.docx]

**S3b Table. Factor loadings obtained with data imputation in the Calibration Sample.**

f1= Childhood/Adolescent Touch Experience; f2 = Comfort with Interpersonal Touch;

f3 = Fondness for Interpersonal Touch; f4 = Adult Touch Experience.

|  | Calibration Sample  (Number of used observations = 1246) | | | |
| --- | --- | --- | --- | --- |
|  | f1 | f2 | f3 | f4 |
| TBQ_01 | 0.739 |  |  |  |
| TBQ_02 | 0.729 |  |  |  |
| TBQ_03 | 0.836 |  |  |  |
| TBQ_04 | 0.660 |  |  |  |
| TBQ_07 | 0.810 |  |  |  |
| TBQ_08 | 0.566 |  |  |  |
| TBQ_15 | 0.702 |  |  |  |
| TBQ_19 | 0.762 |  |  |  |
| TBQ_27 | 0.843 |  |  |  |
| TBQ_28 | 0.635 |  |  |  |
| TBQ_21 |  | 0.712 |  |  |
| TBQ_22 |  | 0.694 |  |  |
| TBQ_23 |  | 0.688 |  |  |
| TBQ_24 |  | 0.784 |  |  |
| TBQ_25 |  | 0.879 |  |  |
| TBQ_26 |  | 0.697 |  |  |
| TBQ_10 |  |  | 0.432 |  |
| TBQ_11 |  |  | 0.814 |  |
| TBQ_12 |  |  | 0.773 |  |
| TBQ_16 |  |  | 0.391 |  |
| TBQ_18 |  |  | 0.428 |  |
| TBQ_05 |  |  |  | 0.690 |
| TBQ_06 |  |  |  | 0.763 |
| TBQ_09 |  |  |  | 0.687 |
| TBQ_13 |  |  |  | 0.787 |
| TBQ_14 |  |  |  | 0.793 |
| TBQ_20 |  |  |  | 0.623 |
| TBQ_29 |  |  |  | 0.605 |
