# Supplementary material for: Tactile Biography Questionnaire: A contribution to its validation in an Italian sample
Source: PLoS One. 2022 Sep 15;17(9):e0274477. doi: 10.1371/journal.pone.0274477 (PMC9477375; doi:10.1371/journal.pone.0274477)
Supplement: S3 Table — a. Fit indices obtained by imputing missing data with full information maximum likelihood approach (film) in the Calibration Sample. CFI = comparative fit index; NNFI = Tucker–Lewis index; RMSEA = root mean square error of approximation; SRMR = Standardized root mean square residual. TCD = total coefficient of determination. b. Factor loadings obtained with data imputation in the Calibration Sample. f1 = Childhood/Adolescent Touch Experience; f2 = Comfort with Interpersonal Touch; f3 = Fondness for Interpersonal Touch; f4 = Adult Touch Experience. (ZIP) [file pone.0274477.s012.zip › S3a_Table.docx]

**S3a Table. Fit indices obtained by imputing missing data with full information maximum likelihood approach (film) in the Calibration Sample.**

CFI = comparative fit index; NNFI = Tucker–Lewis index; RMSEA = root mean square error of approximation; SRMR=Standardized root mean square residual. TCD = total coefficient of determination.

|  | CFI | NNFI | RMSEA | SRMR | TCD |
| --- | --- | --- | --- | --- | --- |
| Calibration Sample | 0.817 | 0.801 | 0.089 | 0.074 | 0.999 |
